# Characteristics and standards of severe sagittal imbalance in adult patients with spinal deformities: a retrospective analysis

**DOI:** 10.1186/s12891-024-07231-5

**Published:** 2024-02-09

**Authors:** Yong-Chan Kim, Kee-Yong Ha, Sung-Min Kim, Xiongjie Li, Dong-Hyun Kim

**Affiliations:** 1 Department of Orthopaedic Surgery, College of Medicine, Kyung Hee University Hospital at Gangdong, 892 Dongnam-ro, Gangdong-gu, 05278 Seoul, South Korea; 2 Department of Orthopaedic Surgery, Graduate School of Medicine, Kyung Hee University, Kyungheedae-ro, Dongdaemun-gu, Seoul, South Korea

**Keywords:** Adult spinal deformity, Degenerative flat back, Dynamic sagittal imbalance, Diagnostic criteria, Severity

## Abstract

**Objective:**

To analyze the characteristics of “severe” dynamic sagittal imbalance (DSI) in patients with adult spinal deformity (ASD) and establish criteria for them.

**Methods:**

We retrospectively analyzed 102 patients with ASD presenting four cardinal signs of lumbar degenerative kyphosis. All patients underwent deformity corrective surgery and were divided into three groups according to the diagnostic criteria based on the Oswestry disability index and dynamic features (△Time_walk_: time until C7 sagittal vertical axis [C7SVA] reaches ≥ 20 cm after the start of walking) of sagittal imbalance. The paravertebral back muscles were analyzed and compared using T2-weighted axial imaging. We performed a statistically time-dependent spinopelvic sagittal parameter analysis of full standing lateral lumbar radiographs. Lumbar flexibility was analyzed using dynamic lateral lumbar radiography.

**Results:**

The patients were classified into the mild (△Time_walk_ ≥ 180 s, 35 patients), moderate (180 s > △Time_walk_ ≥ 30 s, 38 patients), and severe (△Time_walk_ < 30 s, 29 patients) groups. The back muscles in the severe group exhibited a significantly higher signal intensity (533.4 ± 237.5, *p* < 0.05) and larger area of fat infiltration (35.2 ± 5.4, *p* < 0.05) than those in the mild (223.8 ± 67.6/22.9 ± 11.9) and moderate groups (294.4 ± 214.7/21.6 ± 10.6). The analysis of lumbar flexibility revealed significantly lower values in the severe group (5.8° ± 2.5°, *p* < 0.05) than in the mild and moderate groups (14.2° ± 12.4° and 11.4° ± 8.7°, respectively). The severe group had significantly lower lumbar lordosis (LL, 25.1° ± 22.7°, *p* < 0.05) and Pelvic incidence-LL mismatch (PI-LL, 81.5° ± 26.6°, *p* < 0.001) than those of the mild (8.2° ± 16.3°/58.7° ± 18.8°) and moderate (14.3° ± 28.6°/66.8° ± 13.4°) groups. On receiver operating characteristic curve analysis, PI-LL was statistically significant, with an area under the curve of 0.810 (95% confidence interval) when the baseline was set at 75.3°. The severe group had more postoperative complications than the other groups.

**Conclusions:**

Our results suggest the following criteria for severe DSI: C7SVA > 20 cm within 30 s of walking or standing, a rigid lumbar curve < 10° on dynamic lateral radiographs, and a PI-LL mismatch > 75.3°.

**Level of evidence:**

3.

## Background

Prevalence of adult spinal deformities (ASD) has increased in aging global population. Management of ASD can be either surgical or nonsurgical. Since the former can result in recovery of spinal alignment and significant improvement in clinical outcomes, it can be the treatment of choice. However, surgical treatment can lead to several early and late complications [1]. Lonergan et al. [2] reported early postoperative complications after ASD surgery in patients aged 70 years and older. These were mainly medical complications, including postoperative anemia, gastrointestinal problems such as constipation or ileus, and urinary retention. Lapp et al. [3] retrospectively reviewed patients who underwent complex surgery for adult spinal deformity and reported late postoperative complications such as pseudarthrosis, loss of correction, or radiographic degenerative changes proximal or distal to the fusion level.

Degenerative flat back, characterized by sagittal imbalance, is a specific type of ASD that is more common in Asian people [4] and is associated with severe degeneration of the lumbar extensor muscles in most patients, resulting in a stooping posture [5]. Although compensatory mechanisms, such as pelvic retroversion, work to overcome sagittal imbalance, but the compensation has limitations and their application is more difficult during walking. Lee et al. [6] described the dynamic features of sagittal imbalance in degenerative flat backs, defined as dynamic sagittal imbalance (DSI). Yin et al. [7] proposed that changes in the C7 sagittal vertical axis (C7SVA) during walking could be a convenient way to detect the severity and characteristics of DSI and suggested treatment strategies for it. However, in our experience, patients with severe DSI have relatively poor surgical outcomes and more postoperative complications. Nevertheless, diagnostic criteria for sever DSI are vague and related studies are lacking. Therefore, this study aimed to analyze the characteristics of ASD in patients with severe DSI and establish diagnostic criteria for this condition.

## Methods

### Study design

Between 2016 and 2019, 196 patients with ASD and DSI were assessed at our center. Enrollment criteria included severe sagittal imbalance with dynamic features (after the start of walking, the C7SVA gradually increases and becomes larger than 20 cm) and four cardinal signs of lumbar degenerative kyphosis (LDK) [8]. Patients with gait disturbances due to leg length discrepancies or history of lower extremity surgery below the hip joint were excluded.

**Fig. 1:**
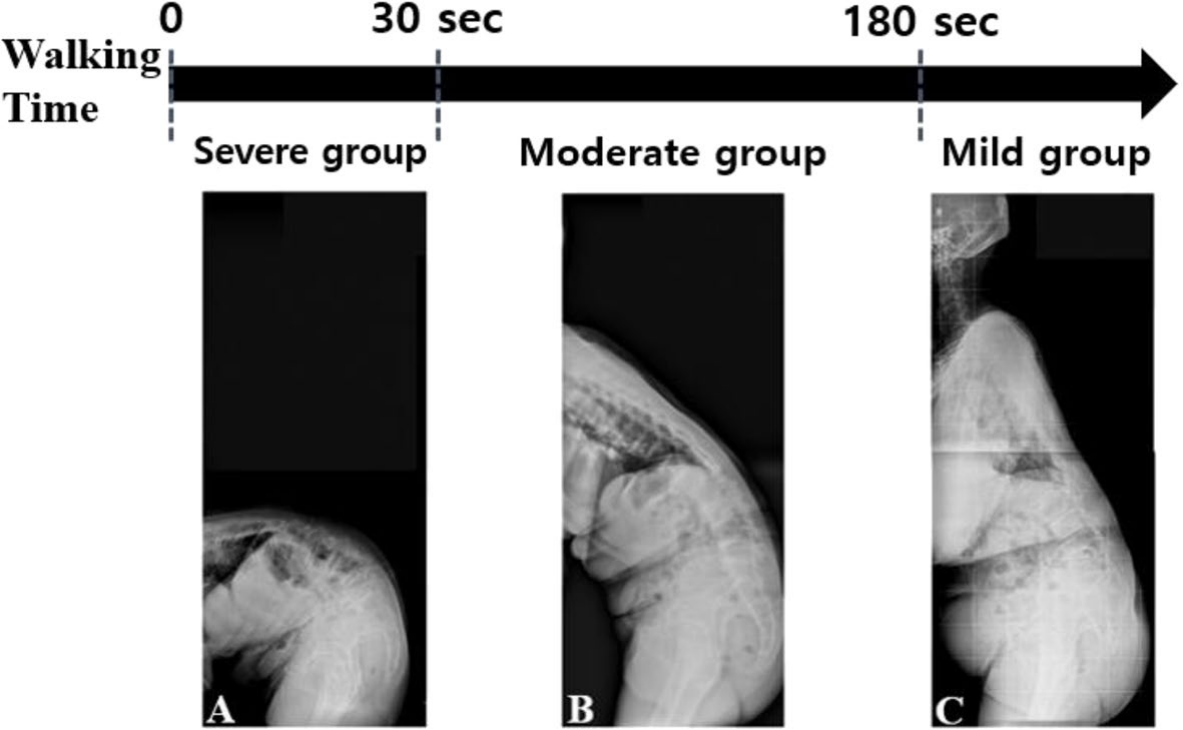
Three groups according to time (△Timewalk) that C7SVA increases after ambulation. All patients were divided into mild (C, △Timewalk ≥ 180 s), moderate (B, 180 s > △Timewalk ≥ 30 s) and severe (A, △Timewalk < 30 s) groups according to the time taken for C7SVA to reach 20cm or more after walking

The 196 patients with DSI were classified into eight subgroups according to evenly divided time frames until C7SVA reached 20 cm or more after the start of walking (△Time_walk_). To determine the minimum clinically important difference (MCID), we calculated the diff_domain_ (MCID deviation of each patient from the normative values) of the preoperative Oswestry Disability Index (ODI) in our patient subgroups [9] (Table 1). We determined the threshold values of DSI [10, 11] and divided the patients into three groups based on severity (Table 2) [12]: mild(△Time_walk_ ≥ 180 s), moderate (180 s > △Time_walk_ ≥ 30 s), and severe (30 s > △Time_walk_) (Fig. 1).

**Table 1:** Preoperative ODI and diff_domain_ of each △Time_walk_ subgroup

	**△Time**_**walk**_** Subgroup**							
	≥ 180 s*	≥ 180 s	150–180 s	120–150 s	90–120 s	60–90 s	30–60 s	0–30 s
**Cases**	50	35	26	18	12	15	9	31
**Proportion**	25.6%	17.8%	13.3%	9.2%	6.1%	7.6%	4.6%	15.8%
**ODI**_**preop**_	16.8 ± 10.5	20.7 ± 18.2	26.1 ± 13.5	29.4 ± 20.1	31.7 ± 17.9	35.8 ± 11.7	39.8 ± 14.5	50.5 ± 12.8
**Diff**_**domain**_	0.972	1.452	2.126	2.539	2.825	3.348	3.848	5.182

*ODI* Oswestry Disability Index, *△Time*_*walk*_  Time until C7SVA reaches 20 cm or more after starting walking, *ODI*_*preop*_  Preoperative ODI, 180 s* Nonoperative group in more than 180 s, *Diff*_*domain*_ MCID deviation of each patient from normative values.  = (ODI_patient domain_ – ODI_normative domain_)/MCID_ODI_

^*^The normative ODI score used in our study was 9.05 points obtained from Tonosu et al.

^*^The MCID value of ODI used in our study was -8 points

**Table 2:** Determination of threshold values of DSI

	**△Time**_**walk**_** Subgroup**			
	≥ 180 s*	** ≥ 180 s** **(Mild group)**	**30 – 180 s** **(Moderate group)**	**0 – 30 s** **(Severe group)**
Diff_domain_ (MCID)	0.972	1.452	2.126 – 3.848	≥ 5.182
Criteria (MCID)	< 1	1 – 2	2 – 4	> 4

*MCID* Minimum clinically importance difference, *Diff*_*domain*_ MCID deviation of each patient from normative value, 180 s^*^ Nonoperative group in more than 180 s

Of the 196 patients, only 102 underwent deformity corrective surgery. The proportion of patients in each group was the following: mild, 34% (35/102); moderate, 37% (38/102); and severe, 29% (29/102). We retrospectively reviewed the medical records of 102 consecutive patients with ASD who had underwent spinal surgery for deformity correction at a single institution between 2016 and 2019. The final follow-up period was two years.

### Data collection

Measurements of △Time_walk_ were recorded under the supervision of a well-trained physician assistant (PA) at our center. Two set of anteroposterior and lateral entire-spine radiographs were requested for patients who underwent deformity corrective surgery. The first set was obtained before walking. The patients were instructed to walk at their usual speed for 10 min without rest, while the second radiograph was obtained immediately afterwards. According to a previous study [6], a 10-min walk was considered sufficient to identify posture changes in an outpatient clinic. The PA supervised each patient during the entire 10-min walk. Patients who were unable to walk for 10 min owing to a stooping posture were examined immediately after the termination of walking, and the time was recorded. For accurate time recording, patients with a C7SVA of 20 cm or more on the postgait radiograph underwent △Time_walk_ measurement again when admitted to the hospital.

The demographic and clinical data included patient age, sex, body mass index (BMI), bone mineral density (BMD: L1–4, T-score), diagnosis including combined pathologies of the lumbar spine, comorbidities, upper instrumented vertebra (UIV), lower instrumented vertebra LIV, operation time (defined as the start to end of anesthesia), blood loss (measured through estimated blood loss), and postoperative complications including intensive care unit (ICU) stay, and proximal junctional kyphosis.

To evaluate back muscle degeneration in each patient, we used a software with picture archiving and communication system. Back muscles included the multifidus and erector spinae of the lumbar region. Using lumbar spine magnetic resonance imaging (MRI), three T2-wighted axial images at each disc level were obtained and evaluated using the midpoint of each level as a reference point. In each MRI data, we evaluated the mean signal intensity, standard deviation (SD) of signal intensity, and fatty infiltration percentage at multiple levels (L1–2, L2–3, L3–4, and L4–5) in the lumbar back muscles were evaluated for each MRI data point [5]. Unlike the study performed by Lee et al. [5], where measured only on the right side, in this study, we measured the cross-sectional area (CSA) of the back muscle compartment on both sides and used averaged value.

Radiologic spinopelvic parameters included C7SVA, thoracic kyphosis (TK, sagittal Cobb angle from the superior endplate of T5 to the inferior endplate of T12), thoracolumbar kyphosis (TLK, sagittal Cobb angle from the superior endplate of T10 to the inferior endplate of L2), lumbar lordosis (LL, sagittal Cobb angle from the inferior endplate of T12 to the superior endplate of S1), pelvic tilt (PT, angle made between lines originating at the bicoxofemoral axis and extending vertically and to the middle of the superior endplate of S1), sacral slope (SS, angle between the superior endplate of S1 and the horizontal line), pelvic incidence (PI, angle between a line perpendicular to the superior endplate of S1 and the line connecting the superior endplate of S1 to the bicoxofemoral axis), proximal junctional angle (PJA: sagittal Cobb angle between the UIV and the UIV plus 2 levels), PI-LL mismatch (PI-LL, mismatch between PI and LL), and lumbar flexibility. Lumbar flexibility was defined as the difference in LL between flexion and extension on lateral radiographs: rigid, < 10º; not rigid, ≥ 10º. In particular, we established a receiver operating characteristic (ROC) curve analysis to determine severity of DSI using PI-LL mismatch values.

Two independent orthopedic spinal surgeons repeated all the measurements after two weeks. The intraclass correlation coefficient (ICC) was measured to assess agreement between the observers [13].

### Statistical analysis

Continuous variables were presented as mean ± SD. Frequency analysis was used to analyze the categorical variables. Analysis of variance and chi-square or Fisher’s exact tests were used, as appropriate, for group comparisons. Statistical significance was set at *p* < 0.05. All statistical analyses were performed using SPSS for Windows (IBM SPSS 21.0, IBM Corp., Armonk, NY, USA). ROC curve analysis was performed using MedCalc software (version 20.1) to assess the specificity, sensitivity, and area under the ROC curve AUC and to select the optimal critical value for PI-LL mismatch.

## Results

### Demographic data

Among the 102 patients who had undergone surgery, the mean age of patients in the severe group was 72.0 years, which was more than those in the mild (68.8 years) and moderate (68.2 years) groups. The groups did not differ significantly in terms of sex ratio or BMI. Regarding BMD, the T-score (L1–4) was -1.87 ± 0.6 in the severe group, which was significantly lower than in the mild (-1.02 ± 0.7) and moderate (-1.25 ± 1.3) groups. Comorbidities of the patients, including hypertension and diabetes mellitus, were similar among the groups. In all groups, UIV was greater than T10 in most cases, and the proportion of patients with UIV greater than T10 was significantly higher in the severe group (100%) than in the other two groups (80% and 94.7%, respectively). Furthermore, operation time (392.3 ± 57.2 min) was significantly longer in the severe group than in the other two groups (mild, 342.2 ± 54.7 min; moderate, 351.6 ± 61.5 min). Additionally, blood loss was significantly greater in patients of the severe group (2,526.7 ± 924.3 mL) than those of the other two groups (mild, 2,213.3 ± 760.5 mL; moderate, 2,253.3 ± 777.2 mL) (Table 3).

**Table 3:** Baseline characteristics of patients in each group

		**Mild** **(*n* = 35)**	**Moderate (*n* = 38)**	**Severe** **(*n* = 29)**	***P* value**
**Age (year)**		68.8 ± 3.99	68.2 ± 4.54	72.0 ± 5.29	0.065
**Sex (female/male)**		32:3	38:0	29:0	0.318
**BMI (kg/m**^**2**^**)**		23.49 ± 3.12	23.48 ± 2.19	22.04 ± 3.51	0.229
**BMD (L1-4, T-score)**		-1.02 ± 1.69	-1.25 ± 1.27	-1.87 ± 0.58	0.038*
**Preop. diagnosis**	**LDK**	2	4	4	0.762
	**Combined state**				
	Spinal stenosis	24 (68.6%)	15 (39.5%)	10 (34.5%)	0.034*
	Spondylolisthesis	1 (2.9%)	1 (2.6%)	2 (6.9%)	0.675
	Old vertebral column fracture	8 (8.6%)	11 (28.9%)	18 (62.1%)	0.025*
	Scoliosis	4 (11.4%)	6 (15.8%)	5 (17.2%)	0.714
	Previous surgery	2 (5.7%)	2 (5.3%)	4 (13.8%)	0.383
**No. of comorbidities**	HTN	14 (40%)	12 (31.6%)	12 (41.4%)	0.726
	Angina	0 (0%)	2 (5.3%)	4 (13.8%)	0.463
	DM	2 (5.7%)	2 (5.3%)	6 (20.9%)	0.154
	CKD	2 (5.7%)	0 (0%)	1 (3.4%)	0.051
	Old CVA	2 (5.7%)	2 (5.3%)	0 (0%)	0.512
	RA	1 (2.9%)	0 (0%)	2 (6.9%)	0.481
	Parkinson’s disease	0 (0%)	2 (5.3%)	0 (0%)	0.714
	COPD	0 (0%)	0 (0%)	2 (6.9%)	0.081
**UIV (n, %)**	≥ T10	28 (80%)	36 (94.7%)	29 (100%)	0.043*
	≤ T11	7 (20%)	2 (5.3%)	0 (0%)	
**LIV (n, %)**	S1	35 (100%)	38 (100%)	29 (100%)	
**Operation time (min)**		342.2 ± 54.7	351.6 ± 61.5	392.3 ± 57.2	0.041*
**Blood loss (ml)**		2213.33 ± 760.51	2253.33 ± 777.24	2526.67 ± 924.32	0.035*

*BMI* Body mass index, *BMD* Bone mineral density, *Preop.* Preoperative, *LDK* Lumbar degenerative kyphosis, *HTN* Hypertension, *DM *Diabetes mellitus, *CKD*  Chronic kidney disease, *CVA* Cerebrovascular accident, *RA* Rheumatoid arthritis, *COPD* Chronic obstructive pulmonary disease, *UIV* Upper instrumented vertebra, *LIV* Lower instrumented vertebra, *min* Minutes, * indicates statistical significance (*p*<0.05)

Strikingly. regarding preoperative diagnosis, LDK, a typical form of degenerative flat back, was rare in all three groups. Most of the patients had one or more pathologies simultaneously. Particularly, the number of patients with spinal stenosis in the mild group (68.6%) and history of vertebral column fracture in the severe group (62.1%) were significantly higher than those in the other groups (Table 3).

### Back muscle degeneration at lumbar levels

The mean signal intensities of the back muscles in the severe group were significantly higher than those in the other two groups at all levels (L1–L2, L2–L3, L3–L4, and L4–L5). The mean signal intensities through the L1–L5 levels were significantly higher in the severe group (533.4 ± 237.5) than that in the other two groups (mild, 223.8 ± 67.6; moderate, 294.4 ± 214.7). The SDs of the signal intensity in the back muscles of patients in the severe group were significantly higher at all levels (L1–L2, L2–L3, L3–L4, and L4–L5). Overall, the fat infiltration area in the back muscles of the severe group was higher, additionally the mean percentage of fat infiltration in L1–L5 levels was significantly higher in the severe group (35.2 ± 5.4%) than in the other two groups (mild, 22.9 ± 11.9%; moderate, 21.6 ± 10.6%). Relative muscle compartment volume was calculated by dividing the CSA of the muscle compartment on the right side by the intervertebral disc area at the same level (muscle CSA/disc CSA). The differences in muscle-disc ratios among the three groups at all levels were not statistically significant (Table 4).

**Table 4:** Signal intensity and fatty infiltration in back muscle

	**Mild** **(*n* = 35)**	**Moderate** **(*n* = 38)**	**Severe** **(*n* = 29)**	***P* value**		
				I vs II	I vs III	II vs III
**Mean of signal intensity**						
L1-L2	204.7 ± 60.9	276.5 ± 205.6	538.2 ± 280.5	0.631	< 0.001*	0.004*
L2-L3	228.0 ± 75.7	299.7 ± 215.3	547.9 ± 289.9	0.657	< 0.001*	0.010*
L3-L4	233.8 ± 75.1	297.4 ± 238.0	499.1 ± 234.8	0.681	0.003*	0.028*
L4-L5	228.9 ± 77.6	303.9 ± 213.9	521.9 ± 233.6	0.556	< 0.001*	0.011*
Mean L1-L5	223.8 ± 67.6	294.4 ± 214.7	533.4 ± 237.5	0.609	0.002*	0.008*
**SD of signal intensity**						
L1-L2	109.1 ± 31.1	168.9 ± 115.9	311.5 ± 158.1	0.369	< 0.001*	0.014*
L2-L3	130.4 ± 45.5	181.8 ± 121.9	316.8 ± 157.2	0.495	0.002*	0.018*
L3-L4	147.2 ± 50.5	185.5 ± 139.8	315.5 ± 138.7	0.673	< 0.001*	0.012*
L4-L5	141.0 ± 43.5	183.3 ± 127.6	306.3 ± 133.7	0.576	< 0.001*	0.014*
Mean L1-L5	131.9 ± 41.3	179.9 ± 124.8	312.5 ± 142.4	0.581	0.003*	0.028*
**Percentage of fat infiltration (%)**						
L1-L2	21.54 ± 15.4	19.3 ± 10.0	31.0 ± 7.1	0.913	0.041*	0.027*
L2-L3	21.0 ± 12.8	21.9 ± 12.2	31.6 ± 6.5	0.972	0.038*	0.043*
L3-L4	25.0 ± 12.0	21.9 ± 13.5	37.1 ± 8.2	0.765	0.022*	0.003*
L4-L5	24.5 ± 12.1	23.3 ± 11.3	41.3 ± 7.2	0.851	0.003*	0.002*
Mean L1-L5	22.9 ± 11.9	21.6 ± 10.6	35.2 ± 5.4	0.873	0.005*	0.002*
**Relative cross-sectional area**						
L1-L2	1.04 ± 0.17	0.95 ± 0.22	0.84 ± 0.30	0.570	0.337	0.440
L2-L3	0.99 ± 0.19	0.92 ± 0.19	0.80 ± 0.27	0.640	0.293	0.315
L3-L4	0.93 ± 0.24	0.83 ± 0.17	0.85 ± 0.26	0.454	0.472	0.955
L4-L5	0.97 ± 0.26	0.77 ± 0.24	0.78 ± 0.15	0.370	0.381	0.932
Mean L1-L5	0.98 ± 0.16	0.87 ± 0.15	0.81 ± 0.23	0.454	0.363	0.560

*L* Lumbar, *SD* Standard deviation, ^*^ indicates statistical significance (*p*<0.05)

### Radiologic parameters and lumbar flexibility

The severe group (12.68° ± 15.23°) had a significantly larger preoperative TLK than that of the other groups (mild, 4.87° ± 7.54°; moderate 6.25° ± 8.65°). Furthermore, patients in this group presented a significantly larger preoperative lumbar kyphosis (25.09° ± 22.73°) compared to those observed in the other groups (mild, 8.21° ± 16.34°; moderate, 14.26° ± 28.58°). In the severe group, pelvic tilt tended to increase, and it was significantly larger (40.22° ± 11.91°) than those in the other two groups (mild, 28.64° ± 9.90°; moderate, 32.13° ± 9.03°). Conversely, the preoperative SS in the severe group was lower than that in the other two groups. The PI-LL mismatch was greater preoperatively in the severe group (81.49° ± 26.59°) than in the mild (58.71° ± 18.84°) and moderate (66.77° ± 13.38°) groups. Preoperative TK and PI were not significantly different among the groups. The lumbar flexibility of patients in the severe group (5.83° ± 2.54°) was significantly lower than those of patients in the other two groups (mild, 14.23° ± 12.38°; moderate, 11.36° ± 8.72°) (Table 5).

**Table 5:** Radiologic parameters & Lumbar flexibility

	**Mild** **(*n* = 35)**	**Moderate (*n* = 38)**	**Severe** **(*n* = 29)**	***P* value**		
				I vs II	I vs III	II vs III
**Preoperative sagittal spinopelvic parameters**						
Thoracic kyphosis (°)	6.36 ± 16.31	5.59 ± 9.73	7.37 ± 11.24	0.884	0.645	0.323
Thoracolumbar kyphosis (°)	4.87 ± 7.54	6.25 ± 8.65	12.68 ± 15.23	0.592	0.007*	0.019*
Lumbar lordosis (°)	8.21 ± 16.34	14.26 ± 28.58	25.09 ± 22.73	0.069	< 0.001*	0.017*
Pelvic tilt (°)	28.64 ± 9.90	32.13 ± 9.03	40.22 ± 11.91	0.213	0.032*	0.061
Sacral slope (°)	22.13 ± 9.77	20.21 ± 14.48	16.05 ± 11.66	0.437	0.021*	0.043*
Pelvic incidence (°)	50.41 ± 13.21	52.47 ± 12.36	56.18 ± 10.68	0.576	0.071	0.281
PI-LL mismatch (°)	58.71 ± 18.84	66.77 ± 13.38	81.49 ± 26.59	0.024*	< 0.001*	< 0.001*
**Immed. LL correction (°)**	59.90 ± 7.47	62.01 ± 9.41	50.28 ± 19.18	0.785	0.031*	0.027*
**Lumbar flexibility (°)**	14.23 ± 12.38	11.36 ± 8.72	5.83 ± 2.54	0.372	< 0.001*	0.032*

*Immed.* Immediate, *LL *Lumbar lordosis, ^*^ indicates statistical significance (*p*<0.05)

Finally, the degree of correction of LL immediately after surgery, was significantly smaller in the severe group (severe, 50.28° ± 19.18°; mild, 59.90° ± 7.47°; moderate, 62.01° ± 9.41°) (Table 5).

### ROC curve analysis

**Fig. 2:**
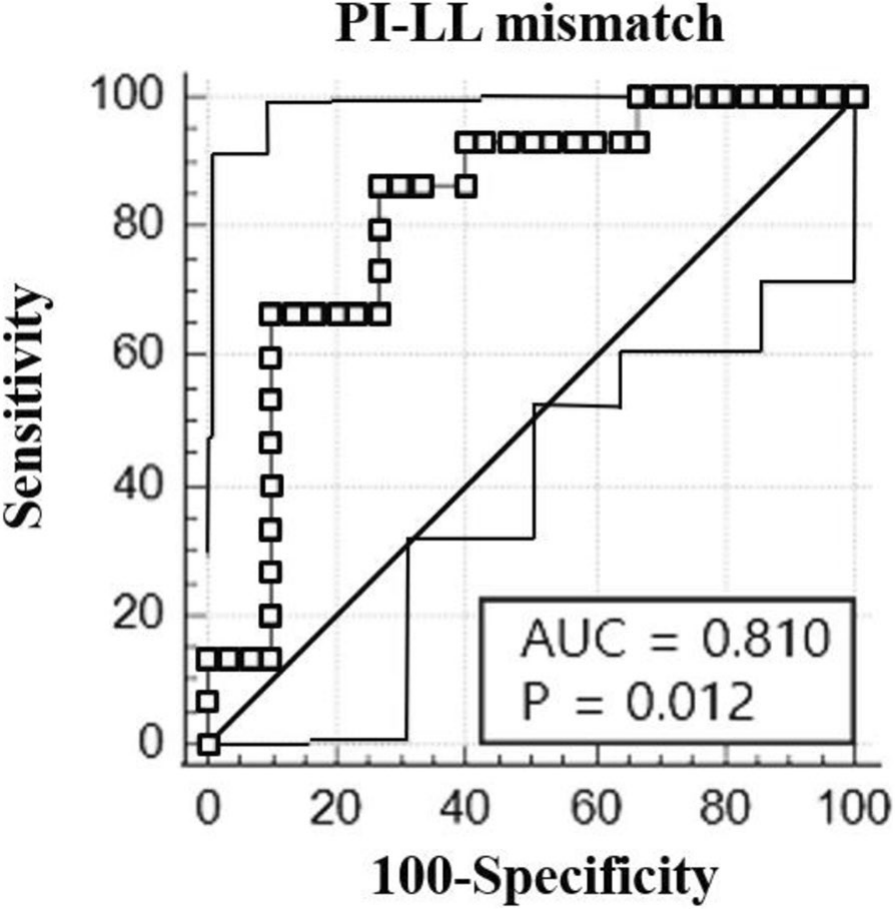
ROC curve analysis of PI-LL mismatch for predicing patient with severe dynamic sagittal imbalance

The AUC of the PI-LL mismatch value, the cutoff point, sensitivity, and specificity in patients in the severe group was 0.810, 75.3°, 66.7%, and 90%, respectively (Fig. 2 and Table 6).

**Table 6:** Receiver Operating Characteristic Analysis of PI-LL mismatch

Parameter	Cut-point	AUROC	Sensitivity	Specificity
PI-LL mismatch (°)	75.3°	0.810 (0.666 – 0.954)	66.67%	90.00%

*AUROC* Area under the receiver operating characteristic curve

### Postoperative complications

After surgery, various complications, such as pseudarthrosis or pneumonia occured. Neurological complications and deep vein thrombosis (DVT) were significantly more frequent in the severe group (13.8% and 13.8%, respectively) than in the other two groups (mild, 0% and 2.6%, respectively; moderate, 0% and 2.6%, respectively). Additional data on complications are presented in Table 7. Furthermore, patients in the severe group (17.2%) experienced more ICU admission than those in the other two groups (mild, 2.9%; moderate, 2.6%) (Table 7).

**Table 7:** Postoperative complications in each group

		**Mild** **(*n* = 35)**	**Moderate** **(*n* = 38)**	**Severe** **(*n* = 29)**	***P* value**
**Complications (n, %)**	PJK	6 (17.1%)	7 (18.4%)	6 (20.7%)	0.129
	Pseudarthrosis	5 (14.3%)	4 (10.5%)	5 (17.2%)	0.621
	Neurological	0 (0%)	1 (2.6%)	4 (13.8%)	0.042*
	Deep vein thrombosis	0 (0%)	1 (2.6%)	4 (13.8%)	0.031*
	Pneumonia	1 (2.9%)	1 (2.6%)	3 (10.3%)	0.077
	Surgical site infection	0 (0%)	2 (5.3%)	0 (0%)	0.745
	Cholecystitis	0 (0%)	1 (2.6%)	3 (10.3%)	0.071
**ICU stay (n, %)**		1 (2.9%)	1 (2.6%)	5 (17.2%)	0.017*

*PJK* Proximal junctional kyphosis, *ICU* Intensive care unit, ^*^ indicates statistical significance (*p*<0.05)

### Assessment of the reliability of measurements using ICC

The ICC values for all measurements showed good-to-excellent inter- and intra-rater reliability, with that of the △Time_walk_ measurements calculated within 0.85 to 0.96. Intra-rater reliability of measurements of back muscle signal intensities, percentage of fat infiltration and relative CSA were 0.88 to 0.97, 0.86 to 0.94, and 0.88 to 0.96, respectively. The intra-rater reliability of the radiologic parameters was 0.87 to 0.96. The ICC for the inter -rater reliabilities of measurements of back muscle signal intensities, percentage of fat infiltration and relative CSA were 0.85 to 0.96, 0.85 to 0.95, and 0.86 to 0.97, respectively. Furthermore, the second measurement (0.84 to 0.96) was more reliable than the first measurement (0.81 to 0.93).

## Discussion

Sagittal imbalance and its dynamic characteristics in patients with degenerative flat backs was studied by Lee et al. [6]. They suggested that the dynamic features of the stooping posture were due to the degeneration of the lumbar extensor muscles and their association with the pelvis and lower extremities. Kim et al. [14] attempted to explain the relationship between dynamic features and pelvic compensation in patients with severe DSI using motion analysis. However, none of the studies so date have clearly defined the criteria or severity of DSI. Yin et al. [7] classified severity according to the change in C7SVA and the resulting ODI value after walking in patients with DSI and presented their own diagnostic criteria. Because the study was conducted on outpatients, few of them showed significant changes in the C7SVA after walking, and their outcomes were good with non-operative treatment. However, patients in the group with a large C7SVA change after walking, the so-called severe DSI group, did not experience symptom relief with non-operative treatment, and most of them underwent surgical treatment. We focused our study in the severe group of patients. In fact, we have encountered many patients with severe DSI uncapable of walking for more 30 s because of stooping, aggravated by walking. Subsequently, they were referred to our hospital for surgical treatment. Therefore, we considered applying walking time to distinguish the severity of DSI.

When comparing demographic data between the groups, there was a significant difference in BMD and posterior fusion segments. To our knowledge, no studies have directly explained the relationship between BMD and severity of sagittal imbalance. In previous studies, osteoporotic compression fracture was reported as a risk factor for sagittal imbalance [15], and we believe that our results are produced in the same context. Additionally, the relatively higher age of patients in the severe group than those of patients in the other groups might have affected the outcomes. Patients in the severe group had relatively higher UIV levels because they required more extensive correction than those required by the other two groups. The operative time and blood loss were significantly higher in the severe group than in the other two groups. This can be inferred from the diagnoses in Table 3 and the pathologies contributing to sagittal imbalance in each group of patients. Patients in the mild group mainly had spinal stenosis, which required multisegment decompression during the surgical procedure. Since patients in the severe group required larger correction, aggressive surgical techniques, such as pedicle subtraction osteotomy (PSO) were required, which could have resulted in longer operation times and larger amounts of blood loss.

Takemitsu et al. [16] suggested that the main pathology of LDK, which is characterized by severe sagittal imbalance, is marked atrophy of the paravertebral muscles accompanied by fatty infiltration. Yagi et al. [4] reported drop-body syndrome as a distinct form of ASD. The criteria included a multifidus CSA < 300 mm^2^, fatty infiltration area > 80%, and normal muscle volume in other areas of body. Additionally, Lee et al. [6] emphasized that the dynamic feature of sagittal imbalance is not a direct effect of skeletal deformity but rather a secondary phenomenon following weakness of the paravertebral muscles. Moreover, many studies have reported that degeneration of the paraspinal back muscle is related to the stooping posture. In our study, paraspinal muscle degeneration was more pronounced in patients in the severe group than in the other groups. This implies that back muscle degeneration not only causes sagittal imbalance but also affects its severity.

Although the topic of our study was related to sagittal imbalance, we did not compare preoperative C7SVA as a sagittal spinopelvic parameter (Table 5). This was because most of our patients had severe stooping and C7 was not visible on the lateral entire-spine radiograph; therefore, it could not be accurately measured and compared. The groups did not differ significantly in terms of PI; PT increased from the mild to the severe group, whereas SS exhibited the opposite trend. In all groups, the lumbar spine showed kyphosis, which was most prominent in the severe group. As the loss of LL increases, the pelvic compensation mechanism for upright posture works more strongly, and SS decreases in the severe group. The increase in the lumbar kyphosis angle among the groups led to a distinct difference in the PI-LL mismatch. Schwab et al. selected the PI-LL mismatch as a sagittal modifiers, set the threshold to less than 10°, and reported its strong correlation with health-related quality of life (HRQOL) in patients with ASD [17, 18]. Therefore, all groups, especially the severe group, might have experienced a lot of discomfort in their daily lives, which explains their decision to undergo corrective surgery without hesitation. In addition, as the PI-LL mismatch was more prominent in the severe group compared to the other two groups, ROC analysis was performed additionally. Consequently, a cut-off value of 75.3° was obtained, which established one criterion for the severe group (Table 6). Understanding the degree of spinal flexibility in patients with sagittal imbalance is important for planning surgery because patients with rigid or fixed deformities require more aggressive surgical procedures, such as osteotomy. Karikari et al. [19] reported that in patients with rigid deformities, satisfactory results were not obtained in radiologic and clinical outcomes when osteotomy was not performed. Sharma et al. [20] compared LL measured on a standing radiograph with LL measured on MRI performed in the supine position and classified it as flexible if the difference was ≥ 10°. However, no widely accepted cut-off value is available for the formula to determine whether the deformity is flexible. We used the difference in LL between flexion and extension on lateral radiographs to evaluate the flexibility of the lumbar spine. The lumbar flexibility of patients in the severe group was significantly lower than those in the other two groups. These results were related to the preoperative diagnoses of our patients, as shown in Table 3. As explained earlier, patients in the mild group had relatively more spinal stenosis with multilevel degenerative disc disease, whereas those in the more severe group had a fixed deformity due to bony changes, such as erosive changes, compression fracture history, or previous operation history; hence, the flexibility might have differed [15]. Therefore, we judged that lumbar flexibility was a reasonable criterion for the severe group and the standard was set at 10°.

In patients with sagittal imbalance, changes in LL and C7SVA are more important than changes in other radiological parameters after surgery. Among the three groups, the degree of LL correction after surgery in the severe group was significantly lower than that in the other two groups (Table 5). Regarding these results, we can consider problems related to “over-correction”. Aggressive procedures such as PSO are frequently used in patients with severe disease who require relatively more extensive correction. Dorward et al. [21] reported many complications that may occur after osteotomy for deformity corrective surgery. In our experience, ICU admission along with a operation time, large blood loss, or a long bed rest period after corrective surgery could affect the degree of correction. Furthermore, flexibility of patients is also related. Patients in the mild or moderate groups were relatively more flexible; therefore, if they were in the prone position under general anesthesia, some correction occurred spontaneously. Therefore, the correction angle may have been reduced during surgery. However, this phenomenon occurred rarely in the severe group. Therefore, for the above reasons, it can be considered that the severe group, which requires a greater degree of correction, showed less correction than the other two groups.

Patients in the severe group, presented a higher probability to experience postoperative complications than those in the other two groups (Table 7). Neurological complications, DVT, and ICU admission rate were significantly higher in the severe group. In 2016, Smith et al. [22] reported that neurological complications occurred in 27.8% of all patients during a minimum 2-year follow-up of surgery for ASD. In our study, 5 out of 102 patients (approximately 5%) experienced neurological complications, and 4 of them belonged to the severe group. All neurological complications were transient and minor and did not require reoperation. We believe that most of these were temporary events that occurred after overcorrection; however, the cause was difficult to predict in some cases. The incidence of DVT after spinal surgery ranges from 0.3% to 31% [23]. In the severe group, 4 out of 29 (approximately 13.8%) experienced DVT. In the severe group, staged operations were performed in almost all cases, and the bed rest period was relatively long compared with those in the other two groups. In the former group, aggressive procedures, such as osteotomy, were performed to obtain a larger correction. Therefore, the incidence of DVT was expected to be high. Schwab et al. [24], through a multicenter review, reported large estimated blood loss (EBL), long hospitalization period, and staged operation as risk factors for major perioperative complications in ASD. In the severe group, five patients were admitted to the ICU. Three of them were transferred for close observation immediately after surgery because their vital signs, such as blood pressure, were temporarily unstable owing to large blood loss during surgery. The other two patients were transferred to the ICU for complications that occurred during hospitalization. One patient was admitted for pneumonia, and the other was for DVT with pulmonary thromboembolism.

This study had limitations in that it was retrospectively conducted in a relatively small numbers, mainly due to the difficulty in recruiting elderly patients with flat back syndrome who desired deformity correction surgery. Because this study was conducted at a single institution on patients who failed long-term conservative treatment and decided to undergo surgical treatment, recall bias may exist. In addition, patient-reported outcomes, such as the scoliosis research society-22, were not compared between the groups. A comparison of the HRQOL outcomes of patients with DSI after surgery may provide a better understanding of characteristics of patients with severe DSI. Despite these limitations, the strength of this study is that it proposes new diagnostic criteria that classify patients based on their dynamic clinical condition, representing the actual discomfort of the patient rather than a specific radiologic parameter, and provides with surgical outcomes of a single institution. Therefore, this study aimed to propose guidelines for the homogeneity of diagnosis, surgical indications, and treatment results in patients with severe flat back syndrome requiring surgical treatment.

## Conclusions

Our results suggest three criteria for severe DSI in adult with spinal deformities: first, C7SVA > 20 cm within 30 s after walking or standing; second, rigid lumbar curve < 10° on dynamic lateral radiographs; and third, PI-LL mismatch > 75.3°.

### Disclosure

The authors report no conflicts of interest concerning the materials or methods used in this study or the findings specified in this paper. No financial support was received for this study.

## Data Availability

The datasets generated and/or analyzed during the current study are not publicly available due to ethical concerns but are available from the corresponding author upon reasonable request.

## Abbreviations

DSI: Dynamic sagittal imbalance
ASD: Adult spinal deformity
C7SVA: C7 sagittal vertical axis
LL: Lumbar lordosis
PI: Pelvic incidence
LDK: Lumbar degenerative kyphosis
MCID: Minimum clinically important difference
ODI: Oswestry disability index
PA: Physician assistant
BMI: Body mass index
BMD: Bone mineral density
UIV: Upper instrumented vertebra
LIV: Lower instrumented vertebra
ICU: Intensive care unit
MRI: Magnetic resonance imaging
SD: Standard deviation
CSA: Cross-sectional area
TK: Thoracic kyphosis
TLK: Thoracolumbar kyphosis
PT: Pelvic tilt
SS: Sacral slope
PJA: Proximal junctional angle
ROC: Receiver operating characteristic
AUC: Area under the ROC curve
DVT: Deep vein thrombosis
PSO: Pedicle subtraction osteotomy
HRQOL: Health-related quality of life
EBL: Estimated blood loss
